# Supplementary material for: Multiplexed Nanometric 3D Tracking of Microbeads Using an FFT-Phasor Algorithm
Source: Biophys J. 2020 Jan 23;118(9):2245–57. doi: 10.1016/j.bpj.2020.01.015 (PMC7202940; doi:10.1016/j.bpj.2020.01.015)
Supplement: Document S1. Figs. S1–S3 [file mmc1.pdf]

Biophysical Journal, Volume *118*

Supplemental Information

Multiplexed Nanometric 3D Tracking of Microbeads Using an FFT-Phasor Algorithm

Thomas B. Brouwer, Nicolaas Hermans, and John van Noort

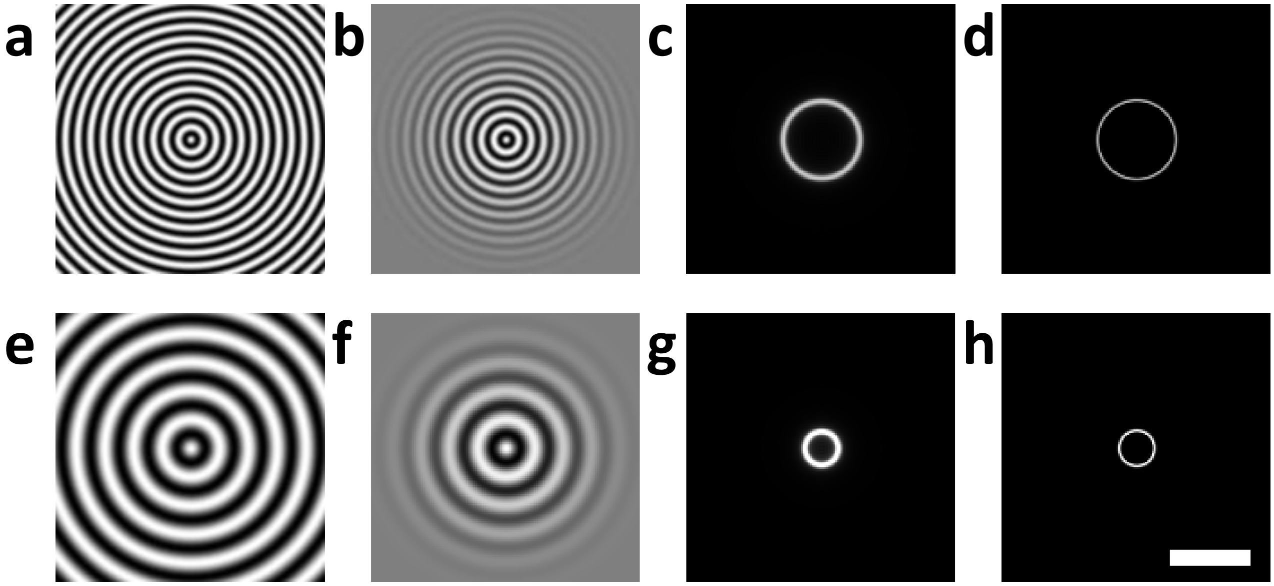

Figure S1) Reference images and corresponding band-pass filters for 3DPT. A reference image containing a single spatial frequency of period $k_A = 7$ pix (a) was filtered by a Hamming filter (b). The reference image was transformed into Fourier space (c), and filtered by a frequency band-pass filter (d). Panel e-h depicted a reference image containing a spatial frequency of period $k_B = 16$ pix. Scale bar: 3 µm.

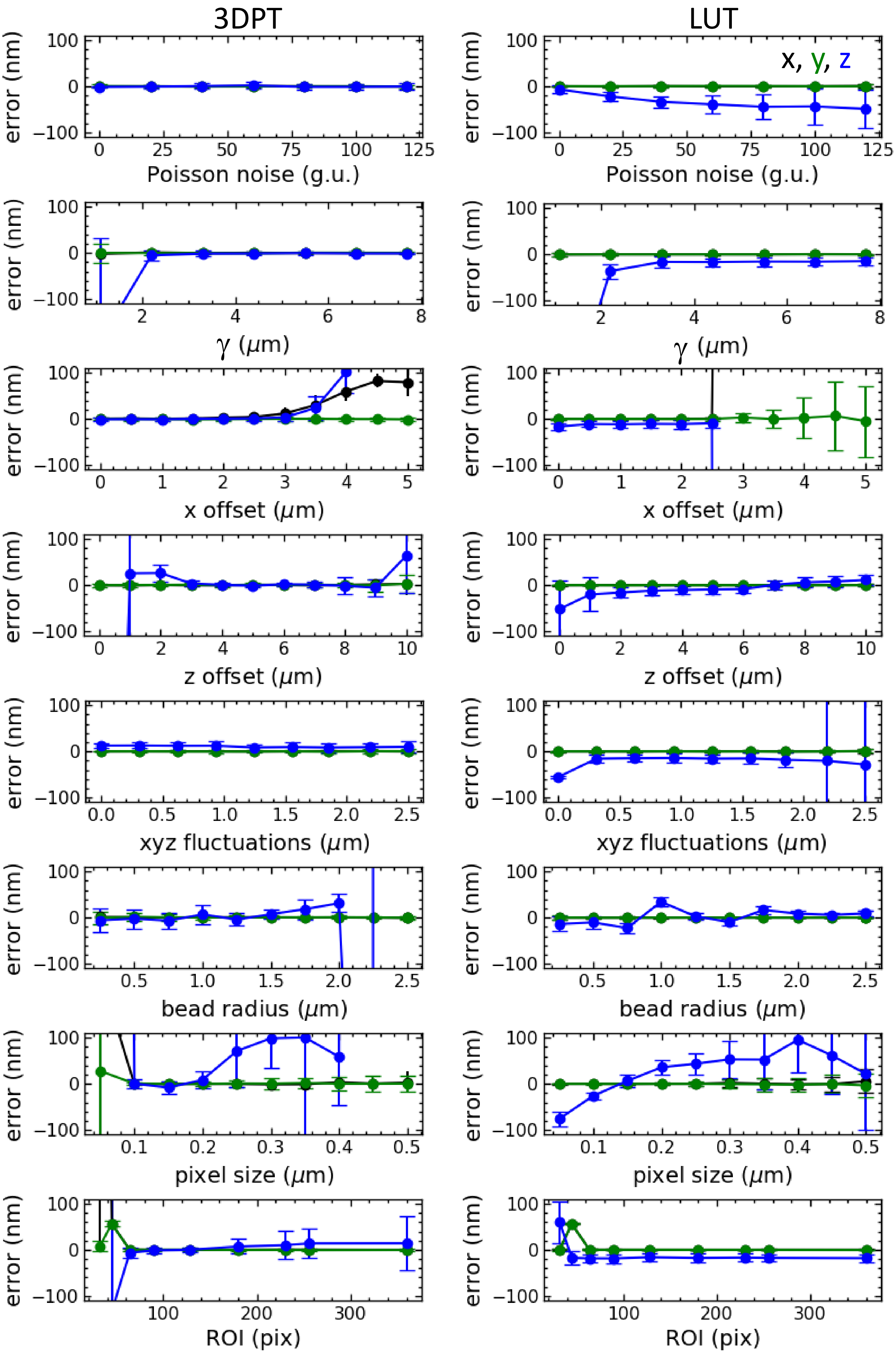

3DPT
LUT
x, y, z
error (nm)
Poisson noise (g.u.)
γ (μm)
x offset (μm)
z offset (μm)
xyz fluctuations (μm)
bead radius (μm)
pixel size (μm)
ROI (pix)

Figure S2) Comparison between 3DPT and LUT accuracy. The accuracies as a function of several parameters that define a tracking experiment were computed using LMST simulations. We used the following standard parameters: ROI size = 100 pixels, pixel size 0.11 µm, number of radial profiles = 64, z-range for calibration was 0 to 15 µm, X and Y offset = 0 µm, Poisson noise = 0 gray scale units. A standard tracking simulation consisted of 256 frames, bead diameter = 1 µm, a linear z-ramp from 4 to 7 µm, X and Y offset = 0 µm, Poisson noise = 5 gray scale units and X, Y, and Z positions that were drawn from a normal distribution with a width of 100 nm. Points represent median difference between input coordinates and computed coordinates. Error bars represent inter quartile ranges of the fluctuations within a tracking simulation. Means and standard deviations were disproportionally affected by rare outliers in the LUT tracking. X and Y data generally overlap and demonstrate the reproducibility of these simulations. Graphs on the left represent 3DPT results, graphs on the right LUT-tracking results.

Poisson noise hardly affected 3DPT results, but introduced a systematically smaller bead height using LUT tracking. Coherence length of the illumination source and NA of the objective were effectively included in the width of the Hamming filter γ, and did not affect tracking accuracy of 3DPT when larger than 2 µm. For LUT, a similar trend was found, but the z coordinate was again underestimated. Offsets in the imaging plane did not affect accuracy up to 3 µm for 3DPT, and was inhibiting LUT tracking. In the z direction, 3DPT was accurate between 3 and 9 µm above focus. Here LUT tracking appeared robust over a larger range, but accuracy was a bit decreased. Simultaneous fluctuations in all 3 directions, mimicking more realistic experiments, did not affect 3DPT accuracy within 2.5 µm, whereas LUT tracking produced large variations in accuracy beyond 2 µm. For bead radii larger than 2 µm we observed reduced accuracy in 3DPT, which resulted from a more intricate diffraction pattern in close vicinity of the bead (data not shown). To remedy this, we computed images at 8 µm above focus, rather than 4 µm, which resulted in less convolved holograms. As a result 3DPT performed well again, up to beads with a radius larger than 2 µm. LUT tracking proved to be more flexible in terms of bead size, though slightly less accurate. Image magnification also affected tracking accuracy for 3DPT. This is not surprising as the spatial frequencies in the holographic image directly scale with magnification. The chosen frequencies were optimized for a magnification corresponding to 0.11 µm per pixel. Larger magnifications yielded poorer accuracy. However, this can be remedied by scaling the spatial frequencies in the reference images accordingly. Somewhat surpassingly, the LUT method also performed less with increasing magnification. This can probably be attributed to the reduced number of fringes that are captured in a fixed sized ROI. Finally, ROI sizes above 64 pixels were sufficient for both methods to yield accurate results. Note however that 3DPT became less accurate for very large ROIs, presumably because of the increased influence of Poisson noise when more pixels are included. Apparently LUT tracking is better at averaging out these fluctuations.

Overall, both methods can achieve similar accuracies over a large range of parameters. 3DPT appeared more robust, yielding < 10 nm accuracy over a broader range of parameters, and featured smaller systematic errors.

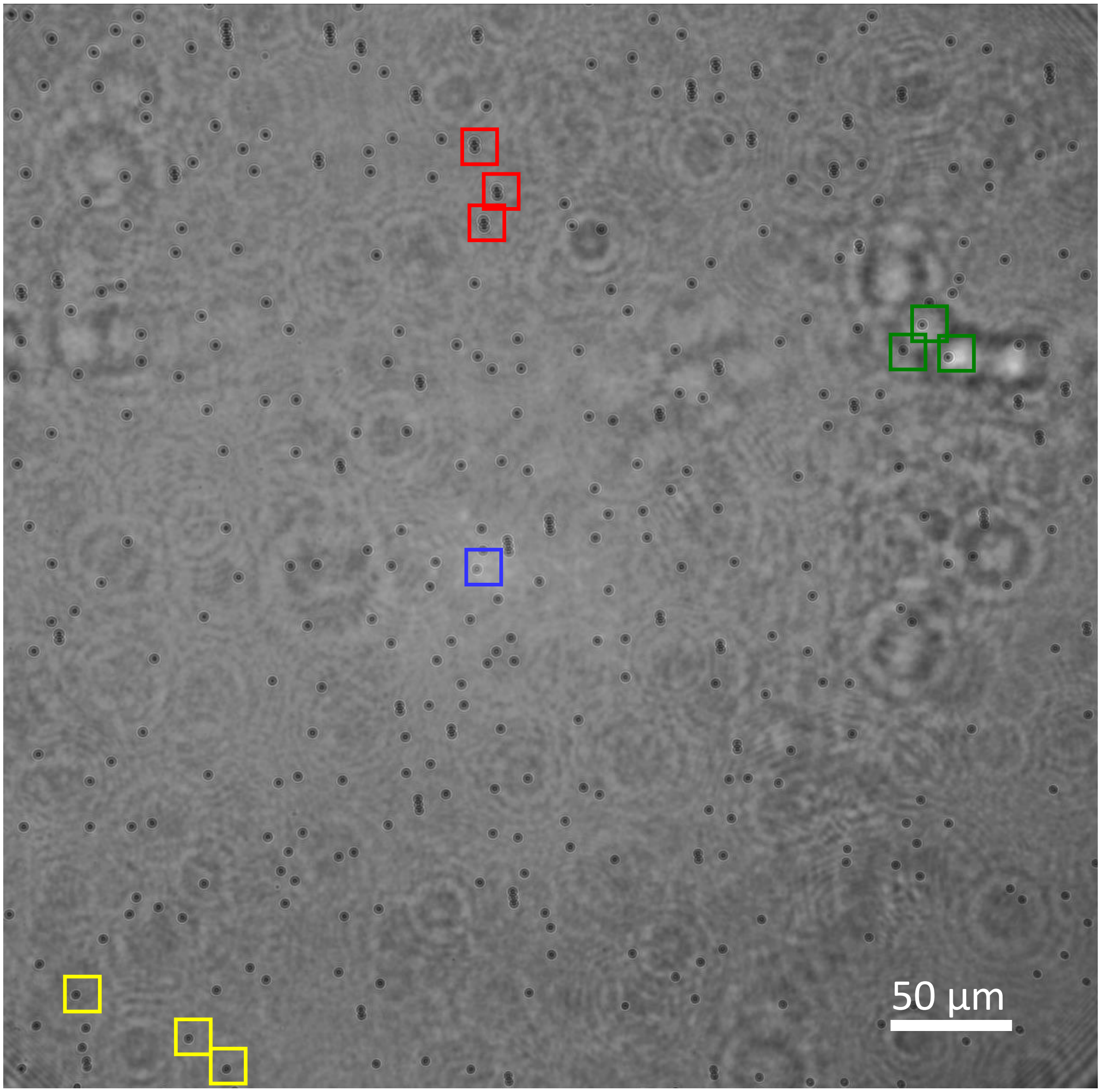

Figure S3) Typical image artefacts in large area imaging. This image shows a full 4096x4096 pixels field of view of tethered beads in our MT microscope. Though holographic images of the beads are well resolved throughout the image, inhomogeneous illumination and image distortions deform the bead images. In particular spherical aberrations dominate at the edges, leading to elongated diffraction patterns (yellow). The center of the field of view was somewhat over exposed, reducing the contrast in the central ROIs (blue). Loose beads, well above the focus plane, obstruct illumination and cause a gradient in the background (green). 3DPT is quite tolerant for such distortions (see figure 5 in the main text). Aggregated beads (red) however, not only affect tracking, but also result in increased forces that are applied to the tether. These beads should be discarded from further analysis. Surprisingly, bead aggregation were not easily identified from 3DPT traces, which were remarkably accurate and error free for small aggregates and should therefore be identified directly in the image. Overall, 3DPT could accurately track all beads in this image.
