# Supplementary material for: Multiplexed Nanometric 3D Tracking of Microbeads Using an FFT-Phasor Algorithm
Source: Biophys J. 2020 Jan 23;118(9):2245–57. doi: 10.1016/j.bpj.2020.01.015 (PMC7202940; doi:10.1016/j.bpj.2020.01.015)
Supplement: Document S2. Article plus Supporting Material [file mmc2.pdf]

Biophysical Journal
Article

Biophysical Society

Multiplexed Nanometric 3D Tracking of Microbeads Using an FFT-Phasor Algorithm

Thomas B. Brouwer,[1] Nicolaas Hermans,[1] and John van Noort[1,*]
[1]Biological and Soft Matter Physics, Huygens-Kamerlingh Onnes Laboratory, Leiden University, Leiden, the Netherlands

ABSTRACT Many single-molecule biophysical techniques rely on nanometric tracking of microbeads to obtain quantitative information about the mechanical properties of biomolecules such as chromatin fibers. Their three-dimensional (3D) position can be resolved by holographic analysis of the diffraction pattern in wide-field imaging. Fitting this diffraction pattern to Lorenz-Mie scattering theory yields the bead's position with nanometer accuracy in three dimensions but is computationally expensive. Real-time multiplexed bead tracking therefore requires a more efficient tracking method, such as comparison with previously measured diffraction patterns, known as look-up tables. Here, we introduce an alternative 3D phasor algorithm that provides robust bead tracking with nanometric localization accuracy in a z range of over 10 μm under nonoptimal imaging conditions. The algorithm is based on a two-dimensional cross correlation using fast Fourier transforms with computer-generated reference images, yielding a processing rate of up to 10,000 regions of interest per second. We implemented the technique in magnetic tweezers and tracked the 3D position of over 100 beads in real time on a generic CPU. The accuracy of 3D phasor tracking was extensively tested and compared to a look-up table approach using Lorenz-Mie simulations, avoiding experimental uncertainties. Its easy implementation, efficiency, and robustness can improve multiplexed biophysical bead-tracking applications, especially when high throughput is required and image artifacts are difficult to avoid.

SIGNIFICANCE Microbeads are often used in biophysical single-molecule manipulation experiments, and accurately tracking their position in three dimensions is key for quantitative analysis. Holographic imaging of these beads allows for multiplexing bead tracking, but image analysis can be a limiting factor. Here, we present a three-dimensional tracking algorithm based on fast Fourier transforms that is fast, has nanometric precision, is more robust against common artifacts than the traditional look-up table method, and is accurate over tens of micrometers. We show its real-time application for magnetic-tweezers-based force spectroscopy on more than 100 chromatin fibers in parallel and anticipate that other bead-based biophysical essays can benefit from this simple and robust three-dimensional phasor algorithm.

INTRODUCTION

Single-molecule techniques overcome ensemble averaging and can resolve unique and rare events at the molecular level (1). By manipulation of microbeads, single-molecule force spectroscopy techniques revealed the mechanical properties of biomolecules such as DNA or RNA with unprecedented detail (2–5). In addition, interactions with proteins such as DNA compaction by histones in eukaryotic chromatin (6–11) and prokaryotic architectural proteins (12–16), supercoiling (17–20), and repair processes (21–23) were extensively studied with magnetic tweezers (MT), optical tweezers (OT), acoustic force spectroscopy (AFS) (24–26), or tethered particle motion (TPM) (13,27,28). These bead manipulation techniques have also been used to quantify the mechanical properties of other biological structures such as extracellular protein collagen (29–31) or even entire cells (32).

The beads not only constitute a micron-sized handle to manipulate the molecules of interest; they also function as a label whose position reflects the extension or deformation of the studied biomolecule. In OT, the position of one or two beads is generally measured from the deflection of a focused laser beam that is projected on a quadrant split detector (33,34), yielding nanometric accuracy and kilohertz bandwidth in three dimensions. MT, TPM, and AFS, however, generally use wide-field imaging with CCD or CMOS cameras and real-time image processing for position measurements. Next to subpixel accuracy, many applications

Submitted September 9, 2019, and accepted for publication January 14, 2020.

*Correspondence: noort@physics.leidenuniv.nl

Editor: Keir Neuman.

https://doi.org/10.1016/j.bpj.2020.01.015

© 2020 Biophysical Society.

This is an open access article under the CC BY license (http://creativecommons.org/licenses/by/4.0/).

require high framerates to resolve fast conformational changes or to capture the full spectrum of thermal motion for accurate force calibration (35). Cameras with kilohertz framerates or tens of megapixel resolution are currently available for fast or large field-of-view imaging (36,37). With such high-end hardware, real-time processing to resolve the three-dimensional (3D) position of the beads becomes rate limiting. Moreover, multiplexing the image processing puts large demands on the processing power of the CPU. In some applications, the GPU is employed to achieve sufficient speed (38–40).

Holographic imaging and subsequent fitting of the images to Lorenz-Mie scattering theory (LMST) has been successfully used to convert videos of colloidal spheres into tracks of 3D coordinates (41). Besides, one can accurately retrieve other physical characteristics that define the hologram, such as the bead radius and refractive index. Despite these advantages, bead-tracking applications that are used in the single-molecule biophysics field generally use simpler, empirical methods to increase processing speed. A popular and fast method for bead tracking splits the tracking into three stages. First, the center of the bead is determined by either computing the center of mass (42–44) or one-dimensional (1D) or two-dimensional (2D) cross correlation with the mirrored intensity profile or predefined kernel (17,34,45–48). Second, a radial intensity profile is computed. Third, this radial profile is compared to a previously calibrated look-up table (LUT) of radial profiles, and the z coordinate is interpolated from the difference curve. Quadrant interpolation minimized cross talk between the x, y, and z coordinates, which significantly increased the tracking accuracy at the cost of being rather computationally intensive (49). Cnossen et al. improved performance by shifting analysis to the GPU, which increased the speed and made it suitable for multiplexed applications. This approach required specialized GPU hardware and advanced software for analysis (50,51).

Previously, we implemented the LUT bead-tracking algorithm in our MT and used it to study the various transitions of chromatin fiber unfolding (6,7,52), as schematically depicted in Fig. 1 *a*. The composition of chromatin fibers may vary because of the quality of reconstitution (11), disassembly (6), or reflecting naturally occurring variations (53). In these applications, we noticed that the dynamic range of the LUT method was sometimes insufficient, yielding an accuracy that depended on the bead height. In our hands, the empirical LUT algorithm frequently flawed because of nonperfect imaging conditions, which led to discarding a large fraction of the beads, limiting the throughput. Tracking errors were enhanced by the increased field of view that is required for imaging multiple beads, which in practice yields more image artifacts such as light gradients due to nonuniform illumination, astigmatism near the edges, or light obstructions by loose beads. Therefore, robustness against common image aberrations becomes increasingly important because the imaging settings can sometimes not be optimized for all beads.

Using the power of 2D fast Fourier transforms (FFTs) to compute cross correlations with computer-generated reference images, we reduced the computational effort to three FFTs and skipped the generation and comparisons with radial profiles, which is the computationally most expensive part of traditional bead tracking. Instead, translations in the z direction were captured into a single parameter, the phase, that we show to be proportional to the height. This, to our knowledge, new tracking algorithm, which we call 3D phasor tracking (3DPT), is simple, sufficiently fast, and robust and meets all criteria for real-time multiplexed nanometric bead-tracking experiments on a generic CPU.

MATERIALS AND METHODS

MT setup

A multiplexed MT setup equipped with a Nikon CFI Plan Fluor objective MRH01401 (NA 1.3, 40×, Oil; Nikon, Tokyo, Japan) was used to track paramagnetic beads. Samples were measured in custom-built flow cells mounted on a multiaxis piezo scanner P-517.3CL (Physik Instrumente KG, Karlsruhe, Germany). A field of view of 0.5 × 0.5 mm^2 was captured on a 25 Mpix Condor camera (cmv5012-F30-S-M-P8; CMOS Vision, Schaffhausen, Switzerland) using an infinity-corrected tube lens ITL200 (Thorlabs, Newton, NJ). The camera was read out by a PCIe-1433 frame grabber (National Instruments, Austin, TX) integrated with a T7610 PC (Dell, Round Rock, TX) equipped with a 10-core Intel Xeon 2.8 GHz processor (E5-2680 v2; Intel, Santa Clara, CA) and 32 GB DDR3 memory. The setup measured the full frame at 30 frames per second. Each pixel measured 112 nm in this configuration. The flow cell was illuminated with a 25 μW 645 nm LED-collimator-packaged (LED-1115-ELC-645-29-2; IMM Photonics GmbH, Unterschleißheim, Germany).

Software

All MT control software, LMST fitting tools, and tracking software were written in LabVIEW (National Instruments). The tracking code and the simulation program are available on Github (https://github.com/JvN2/3DPT).

Spectral analysis of tracking accuracy

A solution of 1 pg/μL 2.8 μm diameter paramagnetic beads was deposited onto a cover slide and heated to 95°C for several minutes to melt the beads to the glass. Subsequently, the cover slide with immobilized beads was mounted into the flow cell and placed onto the setup. Immobilized beads were tracked for 120 s. To obtain σ^2/f_s, the power spectral density (PSD) was calculated and fitted with a horizontal line for $f > 5$ Hz.

LMST

LMST fitting was implemented following (41,54,55). The diffraction pattern $I_{\mathrm{LMST}}(\rho)$ results from the interference between the incident field $E_0(r)$ and the field scattered off the particle $E_s(r)$:

$$I_{\mathrm{LMST}}(\rho) = |E_s(r) + E_0(r)|^2 \big|_{z=0}. \tag{1}$$

The incident field was described by

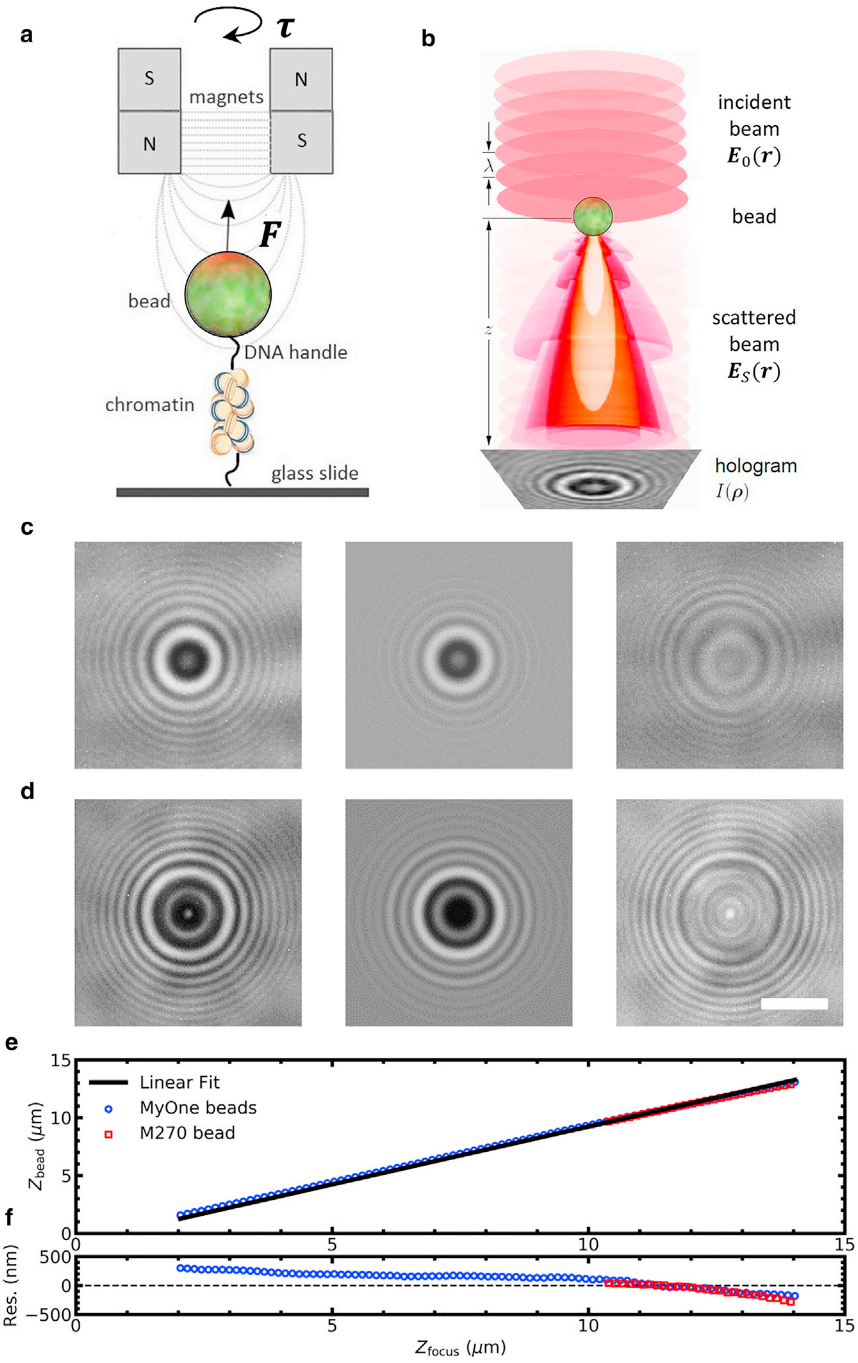

FIGURE 1 LMST cannot fully describe the holographic image of paramagnetic beads used in MT. (*a*) A schematic drawing of a typical MT experiment is given. A molecule tethers a paramagnetic bead to the bottom of a flow cell. The bead is manipulated by a pair of magnets exerting force (***F***) and torque (***τ***). (*b*) A holographic image $I(\boldsymbol{\rho})$ is recorded originating from the interference of an incident beam $\boldsymbol{E}_0(\boldsymbol{r})$ with scattered light $\boldsymbol{E}_s(\boldsymbol{r})$. The diffraction pattern is analyzed to obtain the 3D position of the bead with nanometer accuracy. This image was adapted with permission from Lee et al. (41). (*c*) The diffraction pattern (*left*) of a 1.0 μm diameter paramagnetic bead (Dynabeads MyOne Streptavidin T1; Thermo Fisher Scientific) was fitted with LMST (*center*). The fit to Eq. 5 yielded $x = -70 \pm 1$ nm, $y = 33 \pm 1$ nm, $z = 8300 \pm 100$ nm, $n_{\text{bead}} = 1.9 \pm 0.1$, $\alpha = 0.9 \pm 0.1$, $\beta = 57 \pm 1$, and $\gamma = 57 \pm 1$ (fit $\pm$ standard error). The values $a = 0.5$ μm and $n_{\text{medium}} = 1.33$ were fixed. The residual image (*right*) shows that some features could not be reproduced by LMST. (*d*) The diffraction pattern of a 2.8 μm diameter paramagnetic bead (Dynabeads M270 Streptavidin; Thermo Fisher Scientific) yielded a worse fit: $x = -162 \pm 1$ nm, $y = 84 \pm 1$ nm, $z = 9500 \pm 100$ nm, $n_{\text{bead}} = 1.8 \pm 0.1$, $\alpha = 0.7 \pm 0.1$, $\beta = 49 \pm 1$, and $\gamma = 67 \pm 1$. The values $a = 1.4$ μm and $n_{\text{medium}} = 1.33$ were fixed. Scale bar 3 μm. (*e*) Two paramagnetic beads (MyOne in *blue* and M270 in *red*) were moved through the focus, and the recorded holographic videos were fitted to LMST. For clarity, every fifth data point was plotted. The fits of the diffraction pattern (*black lines*) did not converge close to the focus. Sufficiently far from the focus, the obtained bead height was proportional to z_{focus}. (*f*) Residual of the linear fit of the bead height as a function of the focus height is shown.

$$E_0(\rho) = u_0(\rho)\exp\{ikz\}\hat{\varepsilon}, \tag{2}$$

where the incident field was uniformly polarized in the $\hat{\varepsilon}$-direction so that amplitude $u_0(\rho)$ at position $\rho = (x, y)$ in plane $z = z_p$ of the particle is equal to that in the focal plane $z = 0$. The wavenumber of the propagating wave was $k = 2\pi n_m/\lambda$, where n_m was the refractive index of the medium and λ was the wavelength of the light in vacuum.

The scattered field was described by

$$E_s(\rho) = \alpha \times \exp\{-ikz_p\}u_0(\rho)f_s(\rho - \rho_p), \tag{3}$$

where $f_s(\rho)$ was the LMST function that depended on bead radius a, n_p, n_m, and λ. $\alpha \approx 1$ and accounted for variations in the illumination.

The diffraction pattern was normalized in the $z = 0$ plane by β:

$$\frac{I_{\text{LMST}}(\rho)}{|u_0(\rho)|^2} \equiv \beta \times I_{\text{LMST}}(\rho) = \beta\left[1 + 2\Re\{E_s(r) \times E_0^*(r)\} + |E_s(r)|^2\right]. \tag{4}$$

The normalized image scaled with the calculated Mie scattering pattern $f_s(r)$ by

$$I_{\mathrm{LMST}}(\rho) \approx \beta\left[1 + 2\alpha\Re\left\{f_s(r - r_p) \times \hat{\varepsilon}\exp\{-ikz_p\}\right\} + \alpha^2\left|f_s(r - r_p)\right|^2\right] \times f_\gamma(r), \tag{5}$$

where $f_\gamma(r)$ is a Hamming filter of width γ that represents the decay of the diffraction pattern from the center due to the limited spatial coherence of the light source:

$$f_\gamma(r) = \left(\cos\left(\frac{\pi}{\gamma}r\right) + 1\right)^2. \tag{6}$$

Fitting Eq. 5 yielded the physical parameters a, n_p, n_m, x, y, and z and the scaling coefficients α, β, and γ. The 3D position and radius a of the bead were typically fitted with nanometer precision. The refractive index n_p was reproducible between beads within one part in 1000 (41).

LUT tracking

A standard LUT algorithm was implemented as follows: in both the x and y directions, the central five lines of a region of interest (ROI) were summed. These line traces were correlated with their corresponding mirrored trace. The bead center was assigned to the maximal correlation as interpolated by quadratic fitting. Using this bead center, a radial intensity profile of half the ROI size was computed. The z coordinate was assigned to the minimum of the root mean-square difference with a set of radial profiles that were previously computed for a range of bead heights, again interpolated by fitting a quadratic curve. For comparison with 3DPT, we used the following standard parameters: ROI size = 100 pixels, number of radial profiles = 64, z range for calibration was 0–15 μm, x and y offset = 0 μm, Poisson noise = 0 grayscale units. A standard tracking simulation consisted of 256 frames, we used bead diameter = 1 μm, a linear z-ramp from 4 to 7 μm, x and y offset = 0 μm, Poisson noise = 5 grayscale units, and x, y, and z positions that were drawn from a normal distribution with a width of 100 nm.

3DPT robustness simulations

We simulated videos of beads moving randomly in three dimensions using LMST, with the following parameters: $a = 0.5$ μm, $n_p = 1.9$, $n_m = 1.33$, $\alpha = 1.0$, $\beta = 54$, $\gamma = 45$, and $\lambda = 645$ nm. This approach yielded realistic diffraction patterns of 1.0 μm diameter paramagnetic beads. Parameters n_p, α, β, and γ were average values obtained from fitting the experimental patterns of 186 separate beads (data not shown). For these simulations, the phase was calibrated using 15 polynomials and 15 reference periods. For every simulation, we computed 3600 holograms (150 $\times$ 150 pixels) of beads randomly moving in three dimensions (dx, dy = 0 $\pm$ 5 nm, dz = 12,000 $\pm$ 5 nm). The simulations were equivalent to a 120 s measurement on a 30 Hz camera. The simulated beads were tracked using 3DPT, and the time traces were converted to PSD to extract σ^2/f_s, similar to experimental data.

Five common aberrations were superimposed on the simulated diffraction patterns. Poisson noise was added to the diffraction pattern. Interlacing was simulated by multiplying every other row of pixels with a gain factor. 100% interlacing corresponded to a gain of 2. Light gradients were simulated by adding a slope in the y direction of the diffraction pattern. 100% light gradients corresponded to a curve that rose to a maximal background intensity of 255. Astigmatism was simulated by resampling the columns in the y direction over a smaller number of pixels. 100% astigmatism corresponded to an aspect ratio of 2. Shift out of the ROI was simulated by moving the bead in the x direction until the average bead center was shifted by 50% of the ROI.

Characterization of mechanical vibrations and drift

To characterize 3DPT accuracy experimentally, time traces of 10 immobilized beads were recorded simultaneously. The effects of mechanical vibrations and drift were largely removed by averaging these traces and subtracting it after applying a 1 Hz low-pass filter. The standard deviations (SDs) of the resulting time trace yield the experimental tracking accuracy.

Quantification of the step size of the unwrapping of DNA from the histone core

The length of the stepwise unwrapping of DNA from native nucleosome cores was measured using a method developed by Kaczmarczyk, described in detail in (56). In short, each data point in the force-extension curve was compared to the theoretical extension of a given contour length of free DNA, following a wormlike chain. The theoretical SD for each point was computed using equipartition theorem and the derivative of the force-extension relation of the wormlike chain and supplemented by the tracking error. Next, the z score and corresponding probability that the data point belonged to this contour length were calculated. The probabilities for all data points at a given contour length were summed, and this procedure was iterated for all contour lengths between 0 and the contour length of the DNA substrate. Peaks in the plot of the summed probability as a function of contour length were attributed to a stable state of unfolding of the chromatin fibers, and distances between neighboring peaks reflect single unwrapping events.

RESULTS

Tracking of super-paramagnetic beads using LMST

The scattering of light by colloidal particles and its interference with the incident light is described in LMST. Fig. 1 *b* depicts the contrast mechanism of holographic imaging of a colloidal bead (adapted from (41)). The interference of the incident beam $\boldsymbol{E}_0(\boldsymbol{r})$ with the light that is scattered off the bead $\boldsymbol{E}_s(\boldsymbol{r})$ yields a circularly symmetric hologram $I(\boldsymbol{\rho})$ in the image plane. The center of the hologram corresponds to the xy position of the bead. As the image plane moves away from the location of the bead, the interference pattern expands, leading to more and larger rings around the center of the bead.

We used LMST to fit the six parameters that describe the diffraction pattern: x, y, z, bead radius a, refractive index of the medium n_m, refractive index of the bead n_p, and three additional scaling parameters α, β, and γ (54,55) (see Materials and Methods). We tested two types of super-paramagnetic beads, with a diameter of either 1 μm (Dynabeads MyOne Streptavidin T1; Thermo Fisher Scientific, Waltham, MA; Fig. 1 *c*, *left*) or 2.8 μm (Dynabeads M270 Streptavidin; Thermo Fisher Scientific; Fig. 1 *d*, *left*), that are commonly used in MT. In both cases, we obtained a reasonable fit (Fig. 1, *c* and *d*, *center*), though spherical shapes in the residual images (Fig. 1, *c* and *d*, *right*) indicate that there is a systematic discrepancy between the LMST fit and the experimentally obtained holographic images. The residuals were generally larger for the 2.8 μm beads

than for the 1 μm. Though we did not further investigate this difference, we attribute it to the mixed composition of these beads, which may not fully be captured in the single refraction index used in LMST. Because the 1 μm beads are better described by LMST, we used these smaller beads in the remainder of this work.

For MT force spectroscopy applications, the z coordinate is the most important parameter that can be extracted for the hologram because it quantifies the extension of the tether. In Fig. 1 *e*, the fitted z coordinate is plotted when a bead that was fixed on a cover slide was moved linearly through the focus using a piezo stage. Fitting the obtained diffraction patterns with LMST, a fairly accurate z-position could be obtained over a range of more than 10 μm for the smaller and ~5 μm for the larger bead. The xy coordinates also yielded reproducible results (data not shown). A closer look at the residual of fitting a linear curve to the measured versus applied height, however (Fig. 1 *f*), shows that the systematic errors in the z direction typically amounted to hundreds of nanometers for our paramagnetic beads. The relative error can be smaller for smaller ranges, which makes it still useful for small tethers.

The main limitation of LMST fitting, however, is the processing speed. Fitting the diffraction pattern of a single bead in a 100 $\times$ 100 pixel ROI took several seconds on our CPU. For offline applications, this may not be problematic, although the implementation of offline multiplexed bead tracking would imply storage and processing of very large data files. Real-time processing has the advantage that the experimenter can rapidly assess the quality of the measurements or make adjustments during the measurement. LMST fitting cannot achieve real-time processing with current computing power, which requires a more efficient tracking method.

3DPT

Here, we introduce a novel, to our knowledge, method called 3DPT, which exploits the circular symmetry of the LMST diffraction pattern and its gradual expansion when the bead moves in the z direction to compute bead coordinates. Each step of the tracking algorithm is depicted in Fig. 2. We calculated the xy coordinate by cross correlating the ROI with a computer-generated reference image rather than its mirrored image. The complex reference image $I_n(r)$ resembles the holographic image but consists of a single spatial frequency, characterized by period k_n:

$$I_n(r) = f_s(r) \times \left(\cos\left(2\pi\frac{r}{k_n}\right) + i\sin\left(2\pi\frac{r}{k_n}\right)\right), \quad (7)$$

with r the distance from the center of the ROI of size s. The reference image is spatially filtered with filter $f_s(r)$:

$$\begin{aligned} f_s(r) &= \frac{1}{2}(\cos(2\pi r/s)+1) \quad , r < \frac{s}{2} \\ f_s(r) &= 0 \quad , r > \frac{s}{2} \end{aligned}. \quad (8)$$

Two examples of reference images for two different periods are shown in Fig. 2, *b* and *e*.

The experimentally measured diffraction pattern of the bead I_{bead} is cross correlated with the reference images, yielding cross correlation CC_n:

$$CC_n = \text{FFT}^{-1}(f_B(k) \times \text{FFT}(I_n)^* \times \text{FFT}(I_{\text{bead}})), \quad (9)$$

where a bandpass filter $f_B(k)$ is used in the frequency domain:

$$f_B(k) = \exp\left(-\frac{(k-k_n)^2}{2w^2}\right). \quad (10)$$

The computer-generated images of the reference signal and its filters are depicted in Fig. S1.

Fig. 2, *c–g* show the amplitude and phase images for of the cross correlation with a typical hologram, shown in Fig. 2 *a*, for periods $k_1 = 7$ pixels and $k_2 = 16$ pixels. The amplitude image of the CC_n in both cases featured a single peak that represents the shift of the bead relative to the center of the image. Two amplitude images obtained by cross correlating with reference images with two different periods were multiplied, yielding a sharper peak at the xy position of the bead (Fig. 2 *h*), which was measured with subpixel accuracy using polynomial interpolation in two dimensions.

The z position of the bead was obtained from the phase φ_n at the xy position of the bead. The phase images featured concentric rings around the bead center. In Fig. 2 *i*, we plotted the phase at the bead center of a fixed bead that was moved through the focus in the z direction. Several micrometers above the focus, we obtained good cross correlations with distinct peaks at the bead position. The phase was computed from the amplitude-weighted average of a 10-pixel ROI around the maximum in the amplitude image. The resulting phase increased proportional to the bead height but was wrapped between $-\pi$ and π. Unwrapping of the phase was trivial because the height of the bead increased linearly in time. The phase signal could thus be unwrapped unambiguously, as shown in Fig. 2 *i*. We quantified this curve by fitting a polynomial function to the experimental $\varphi_n(z)$ data, which were inverted to compute the bead height as a function of the measured phase, $z(\varphi_n)$, during subsequent experiments.

Far from the focus, φ_n increased, to a good approximation, linearly with z. Empirically, we found for sufficiently defocused beads

$$\varphi_n(z) \approx c\, k_n^2\, z, \quad (11)$$

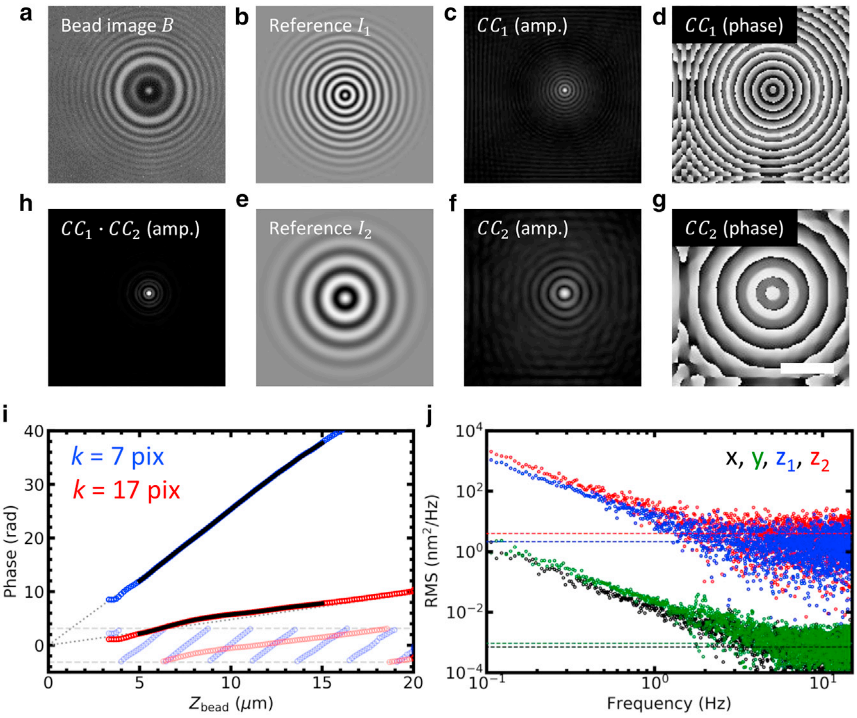

FIGURE 2 The principle of 3D phasor tracking (3DPT). The holographic image (*a*) was cross correlated with two complex reference images I_1 (*b*) and I_2 (*e*), computed using Eq. 7 for period $k_1 = 7$ pixels and $k_2 = 16$ pixels. The cross correlation yielded two complex images CC_1 and CC_2, displayed as amplitude (*c* and *f*) and phase (*d* and *g*). The amplitudes of CC_1 and CC_2 were multiplied, resulting in a sharp peak at the *xy* position of the bead (*h*). The phases at the peak, φ_1 and φ_2, scaled approximately linearly with bead height *z*. Scale bar 3 μm. (*i*) The relation between φ and *z* was calibrated a priori using a measurement in which the focus is linearly shifted in time resulting in phases φ_1 (*semitransparent blue circles*) and φ_2 (*semitransparent red circles*). Subsequently, φ_1 and φ_2 were phase unwrapped, eliminating 2π phase jumps (*blue* and *red* circles beyond the horizontal *dashed lines*). A linear increase in phase starting at the focus roughly describes the phase-height relation (*gray dotted lines*). The unwrapped phases $\varphi_1(z)$ and $\varphi_2(z)$ were fitted with a polynomial, starting 5 μm above the focus (*black lines*). For clarity, every fifth data point was plotted. (*j*) The tracking accuracy of 3DPT was calculated by spectral analysis, yielding an accuracy of 4 $\pm$ 1 nm^2/Hz for z_1 and 2 $\pm$ 1 nm^2/Hz for z_2. The accuracy in *x* and *y* was several orders of magnitude smaller: 0.7 $\times$ 10^{-3} $\pm$ 0.1 nm^2/Hz for *x* and 0.9 $\times$ 10^{-3} $\pm$ 0.1 nm^2/Hz for *y*.

in which *c* represents a calibration factor that depends on the magnification and the refraction index of the immersion medium and did not change between experiments or bead sizes. This linear approximation is plotted in Fig. 2 *i* as dotted lines, and it can be seen that deviations become larger near the focus and for larger periods, as near-field effects of the light scattering become more prominent.

In tracking experiments in which there is no prior knowledge of the bead position, a single phase cannot be converted unambiguously into a unique *z* position because of phase wrapping. A common solution to this phase-unwrapping problem is the use of multiple frequencies (57–60), which was implemented by computing at least two CC_n images with different periods in the reference images. Provisional *z* coordinates, which corresponded to the expected *z* range of the bead spaced by 2π in phase, were calculated. The set of *z* coordinates corresponding to different spatial frequencies that showed the smallest variation was then selected and averaged to compute the final *z* coordinate. Thus, by computing two or more reference images and subsequent cross correlation with an experimental holographic image, it was possible to determine the 3D position of the bead unambiguously.

Performance of 3DPT

The performance of the novel, to our knowledge, 3DPT method was tested in multiple ways. First, the PSD of a time trace of an immobilized bead was computed (see Materials and Methods), as shown in Fig. 2 *j*. The tracking accuracy, expressed in σ^2/f_s, was determined as the plateau value of the PSD at frequencies over 2 Hz (33) because thermal drift and mechanical vibrations introduced low-frequency fluctuations that resulted in increased amplitudes at smaller frequencies (1/*f* noise). The reference image with the highest spatial frequency ($k = 7$ pixels) performed best: $\sigma_z^2/f = 2$ nm^2/Hz. For the lower spatial frequency ($k = 16$ pixels), we obtained an accuracy of $\sigma_z^2/f = 4$ nm^2/Hz. The tracking accuracy in the *x* and *y* directions was several orders of magnitude higher: $\sigma_x^2/f = 0.7 \times 10^{-3}$ nm^2/Hz and $\sigma_y^2/f = 0.9 \times 10^{-3}$ nm^2/Hz. Thus, for a typical framerate of 30 Hz, we can expect a tracking accuracy of 0.2, 0.2, and 10 nm for the *x*, *y*, and *z* coordinates after cross correlation with a single reference image.

Next, to illustrate the increased performance with an increased number of reference images, we plotted the phase calibration graphs obtained for four reference images, shown in Fig. 3. The $\varphi_n(z)$ curve converged to a single point following Eq. 11, which allowed us to unequivocally assign the focus offset. The deviations from linearity, starting 5 μm above the focus, were fitted with a fifth-order polynomial over a range of 10 μm. The residuals of the fits give a good estimation of the dynamic range: the SD, σ_{res}, was generally below 15 nm, as depicted in Fig. 3 *b*. We systematically tested the dependence of the accuracy of the 3DPT method by evaluating the SD of the residuals as a function of the reference frequency. The lowest σ_{res} was found for

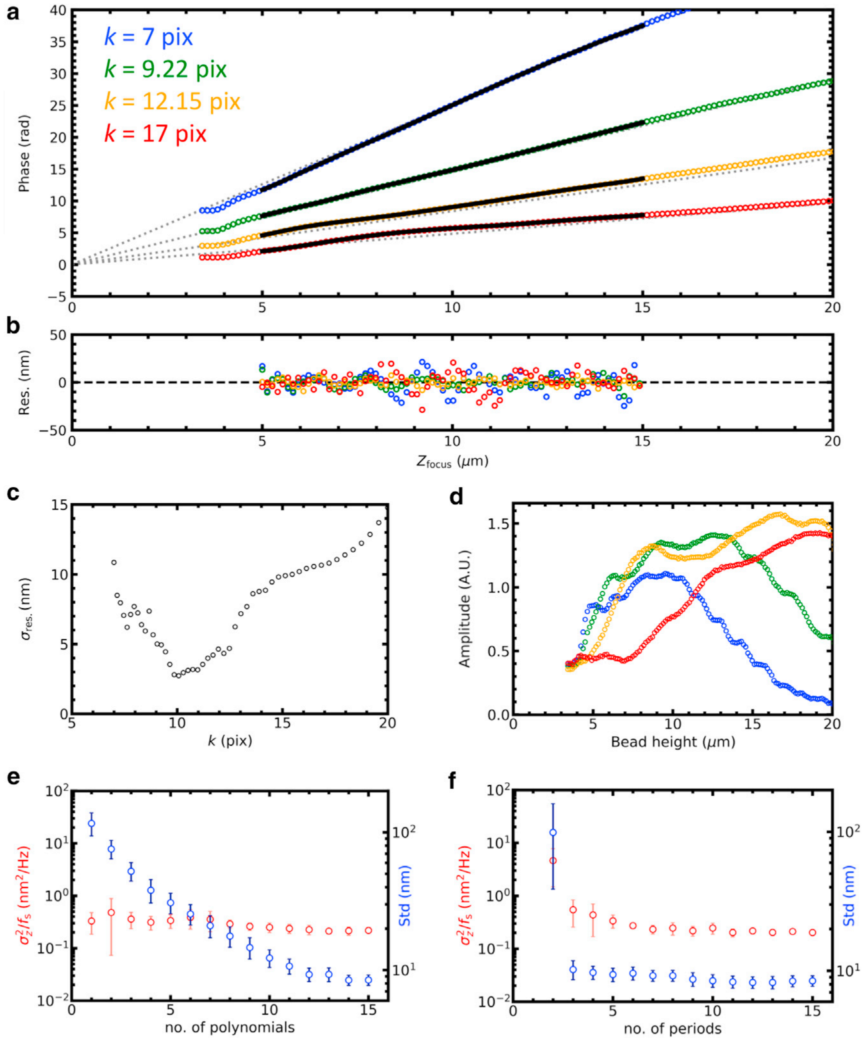

FIGURE 3 Multiple reference images increase the tracking accuracy. (*a*) Phase calibration with four reference images (*colored circles*) is shown. k_n was logarithmically sampled between 7 and 16 pixels. The curves were fitted with a fifth-order polynomial over a range of 10 μm (*black lines*). All curves converged in focus and approximated a straight line (*gray dotted lines*). (*b*) The residuals of the polynomial fit for each reference image are shown (*colored circles*). (*c*) The tracking accuracy obtained with a single reference image varied with its period is shown. The best accuracy was obtained for $k \approx 10$ pixels. Below $k = 7$ pix, the correlation did not yield a distinct peak. (*d*) The variation of the amplitude of the cross correlation with z and k is shown. Larger periods were more prominent as the bead was shifted further out of focus. For clarity, every fifth point was plotted in the graphs (*a*, *b*, and *d*). (*e*) The tracking accuracy in the z direction (*red dots*) slightly increased with the number of polynomials used during phase calibration. The errors significantly reduced up to ~5 polynomials. The average SD of the residuals of the polynomial fit (*blue dots*) linearly decreased up to ~10 polynomials. (*f*) The tracking accuracy in the z direction (*red dots*) increased significantly with the number of reference images, up to ~10 reference images. The use of three instead of two reference images especially was effective: approximately a 10-fold increase in accuracy was established. 15 polynomials were used during phase calibration. The average SD of the residuals of the polynomial fit (*blue dots*) was not affected by the number of reference images when three or more images were used. The errors depicted in (*e*) and (*f*) indicated the SD of six independent beads.

$k = 10$ pixels. For $k < 7$ pixels, we did not obtain distinct correlation peaks, reflecting the diffraction-limited character of the holographic images. For $k > 10$ pixels, σ_{res} gradually increased up to 15 nm, which is still rather accurate for a dynamic range of 10 μm (Fig. 3 *c*). As could be expected from visual inspection of the holographic images, the further the bead was defocused, the less high-frequency information was obtained. This was reflected by the amplitudes of the cross correlations that were plotted in Fig. 3 *d*. Whereas for $k = 7$ pixels, the highest cross correlation was obtained 10 μm above the focus and vanished at 20 μm, larger periods peaked further from the focus. From Fig. 3, *a*–*d*, it is clear that the dynamic range is not limited to 10 μm.

The periodic modulation in Fig. 3 *b* suggests that better accuracy could be obtained by fitting higher-order polynomials. Increasing the order of the polynomials did decrease the average SD of the residuals of the polynomial fit, although this was not reflected in the PSD analysis of the experimental data (shown in Fig. 3 *e*). This is probably due to the slow fluctuations that remain in the residual of the polynomial fit, which would be represented in the low-frequency part of the power spectrum.

The tracking accuracy in the z direction could be increased by combining more reference images at the cost of increased computational time. Fig. 3 *f* shows that the accuracy, as quantified from the PSD, increased ~10-fold when more reference images were used. The accuracy converged to $\sigma_z^2/f = 0.2$ nm^2/Hz for $n = 5$, implying 2.4 nm accuracy for 30 Hz imaging or 1 nm at 5 Hz. The average SD of the residuals of the polynomial fit decreased similarly. For accuracy in the x and y directions, it was sufficient to use more than two reference images to achieve subnanometer accuracy at 30 Hz.

The computation speed was up to 10,000 diffraction patterns per second for 100 × 100 pixel ROIs, even when four reference images were used. Note that the FFT of the reference images can be done before the tracking, so only $(n + 1)$ FFTs need to be computed in real time for n reference images. Subsequent processing (i.e., calculating the xy

position, computing the z coordinate from the calculated phase, and unwrapping the phase) was much faster. Thus, 3DPT is a versatile, accurate, and fast method for holographic tracking of microbeads with nanometer accuracy.

Comparison with LUT tracking

In the most commonly used algorithm for camera-based bead tracking, the height is interpolated from an LUT of radial profiles that were precalibrated for a range of known bead heights. This provides a large increase of the processing rate as compared to LMST fitting. 3DPT is generally slower than LUT tracking because 2D FFTs scale with an ROI size of N pixels as N^2 ln N, and multiple 2D FFTs are required. LUT tracking, on the other hand, uses 1D FFTs that scale as N ln N for xy positioning and calculation of the radial profile scales with N^2. In Fig. 4 *a*, we compared the computation speed of 3DPT with a basic LUT-based tracking algorithm. Indeed, 3DPT is generally slower, and computation time rises faster with larger ROIs. Because the accuracy increases with the number of reference images, it is important to also quantify how this impacts the speed of the calculation. As expected, the time per ROI increases linearly with the number of reference images; see Fig. 3 *b*. For relevant ROI sizes, between 64 and 128 pixels, and five reference images, the processing time is less than 3 ms/ROI, which is slower than the LUT method but sufficiently fast for real-time processing.

We tested both algorithms for accuracy using a set of typical imaging parameters (see Materials and Methods) by computing the difference between the input and output coordinates. Fig. S2 shows, for example, that Poisson noise in the image hardly affected the accuracy and reproducibility of the x, y, and z coordinates for 3DPT but introduced a systematic error and increased variations for the z coordinate in LUT tracking. Note that the error in z did not appear to be the result of inaccurate determination of the center of the bead because x and y accuracy did not change with increasing image noise. Only a small fraction of the LUT coordinates (typically a few percent) featured large outliers, originating from wrongly assigned x, y coordinates. The simulated standard conditions did not include the extreme excursions of the bead or image distortions that happen frequently in experiments, so these errors can only be attributed to the limited range of validity of the 1D x, y tracking. More advanced LUT-tracking schemes (49) may alleviate this problem at the cost of computational speed. Here, we avoid the large impact of these outliers by presenting the tracking error in terms of median ± interquartile range rather than average ± SD. For 3DPT, we did not observe such outliers.

A large number of factors affect the tracking accuracy, and these cannot always be varied in a systematic manner experimentally. Moreover, despite best efforts, it is hardly possible to exclude small variations in mechanical stability, bead size, illumination intensity, and optical aberrations, which can have a major effect on the reproducibility of the tracking data. We therefore used LM theory to evaluate the effect of a number of parameters on the accuracy of both methods. Fig. S2 shows the results of these simulations. Generally, both methods yielded accuracy and reproducibility in the nanometer range, though 3DPT generally performed more accurately and yielded fewer fluctuations. x and y coordinates were more precise than the z coordinate. Changing ROI sizes between 64 and 256 pixels hardly affected accuracy. Differences in magnification, represented by pixel size, did change the accuracy of 3DPT. However, this is expected because spatial frequencies in the image change with magnification, and these should be responded to by adjusting the chosen frequencies in 3DPT accordingly. When correctly chosen, tracking errors reduced to the nanometer range (data not shown). Changes of the coherence length of the illumination source and numerical aperture of the objective were not explicitly included in LMST. We approximated the combined effect of reducing these by applying a Hamming filter over the holographic image (Eq. 6), resulting in a reduction of the number of diffraction rings. Images in which the width of this filter exceeded 2 μm yielded equally accurate results. Overall, this systematic comparison between LUT tracking and 3DPT shows improved accuracy and reproducibility for the latter under typical imaging conditions, at the cost of a two to five times increase in computation time.

The robustness of 3DPT

Large field-of-view imaging, which is a prerequisite for highly parallel tracking of multiple beads, often comes with image artifacts because the entire image may not be

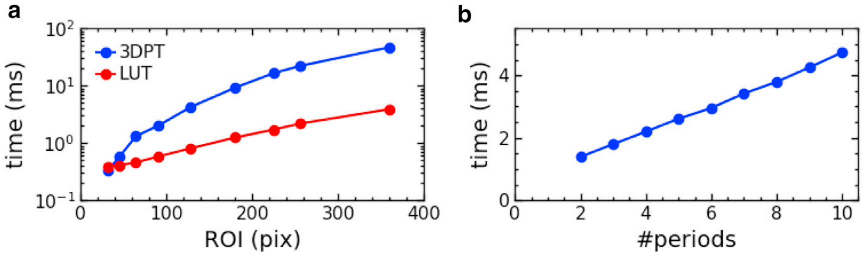

FIGURE 4 3DPT can be computed within several milliseconds per ROI for typical imaging conditions. (*a*) Computation time increases rapidly with ROI size for 3DPT because of multiple 2D FFT computations. LUT tracking is generally several times faster. However, for realistic ROI sizes between 64 and 128 pixels, the processing time is less than 3 ms. These results were computed for five reference images in 3DPT and 64 radial profiles for LUT tracking. (*b*) The time per ROI increases linearly with the number of reference images.

illuminated or imaged optimally. Fig. S3 shows a typical field of view of our setup and highlights such artifacts. To evaluate the robustness of our tracking algorithm for common imaging artifacts, we simulated test data using LMST for 1.0 μm diameter beads that were moving randomly in three dimensions. We simulated the diffraction patterns 12 μm above the focus, at which the accuracy of LMST was the highest (Fig. 1 *f*). This approach allowed us to systematically introduce distortions and to compare tracking results with the known coordinates that were inserted into the LMST.

Fig. 5 *a* shows a simulated image in which we have introduced Poisson noise, representing the signal/noise ratio of a typical camera. Although the accuracy of the *x* and *y* coordinates was hardly affected and remained constant at 0.5 nm^2/Hz, the accuracy in the *z* direction decreased from 0.05 to 10 nm^2/Hz when the noise increased for 0–20 grayscale units in an 8-bit image. Typical experimental noise intensities ($\sim$3 grayscale units) resulted in 1 nm^2/Hz accuracy in the *z* direction, close to the experimentally obtained accuracy in Fig. 3, *e* and *f*.

The robustness of the algorithm was further tested in Fig. 5, *b*–*e*, in which we simulated several other distortions that are frequently observed in experimental imaging. Interlacing, which was prominent in analog CCD cameras, did not affect the tracking accuracy (Fig. 5 *b*). Distortions

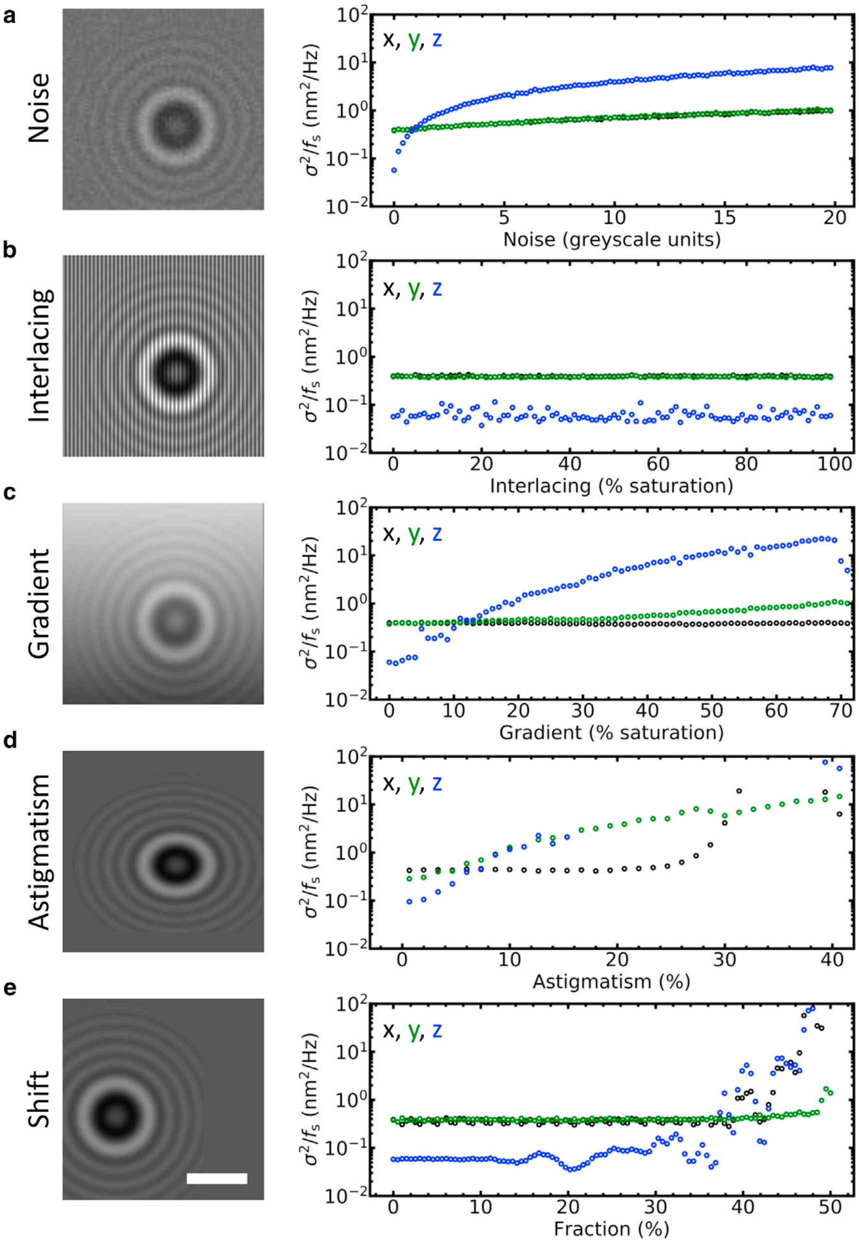

FIGURE 5 3DPT is robust against image aberrations. To evaluate the robustness of 3DPT, the diffraction patterns of 1.0 μm diameter paramagnetic beads were simulated with LMST. In the absence of image artifacts, the tracking accuracy of simulated images was 60 $\times$ 10^{-3} nm^2/Hz in the *z* direction. The diffraction patterns were superimposed with aberrations typically observed in MT and other bead-tracking techniques. Note that the amplitude of the PSD remained below 100 nm^2/Hz, corresponding to 55 nm (0.5 pix) at a framerate of 30 Hz, in all cases except for astigmatism exceeding 30%. Typical experimental values for our microscope were $\sim$3 grayscale units for Poisson noise, 0% for interlacing, $\sim$15% for light gradients, $\sim$2% for astigmatism, and $\sim$5% for shift. (*a*) Poisson image noise mainly affected the accuracy of the *z* coordinate and resulted in a 10-fold decrease in accuracy for an amplitude of 20 grayscale units. The tracking accuracy in the *x* and *y* directions only decreased by a factor of 2. (*b*) Interlacing did not affect the tracking accuracy. (*c*) A light gradient in the ROI up to 20% still yielded an accuracy in the *z* direction below 1 nm^2/Hz. The tracking accuracy was only slightly affected in the *y* direction, the direction of the light gradient, whereas the *x* direction was unaffected. When the light gradient exceeded 70%, no correlation peak was found. (*d*) Astigmatism significantly affected tracking accuracy in three dimensions. (*e*) Our tracking method was unaffected when the bead center was shifted less than 30% out of the ROI. Exceeding a shift of $\sim$40% resulted in the complete loss of tracking in both the *z* direction and the *x* direction (the direction that the bead was moving). Scale bars, 3 μm.

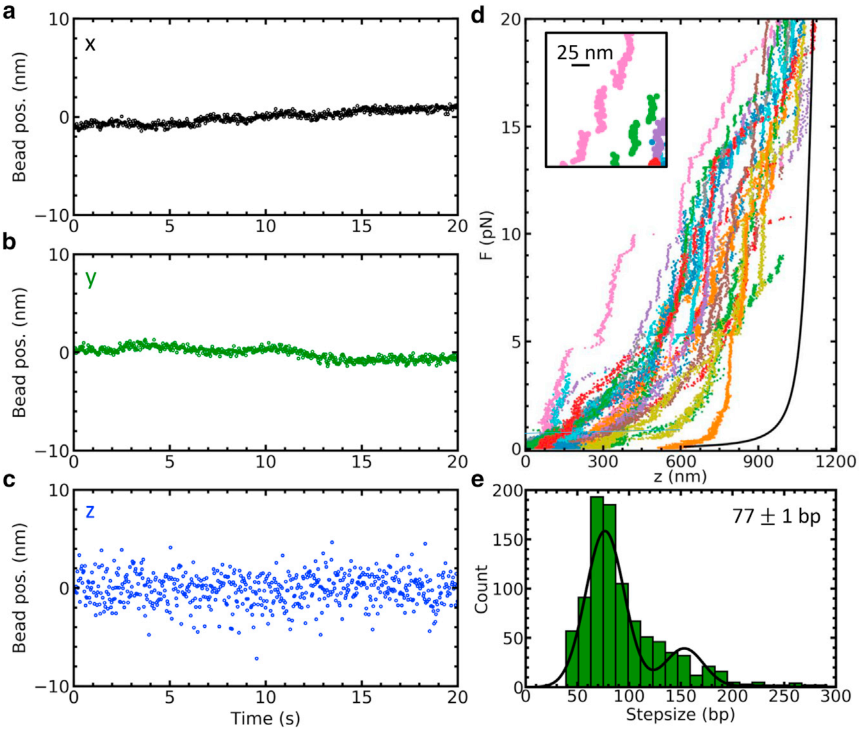

FIGURE 6 Simultaneous nanometric tracking of multiple paramagnetic beads yields nanometer accuracy in three dimensions. (*a–c*) 10 immobilized 2.8 μm diameter paramagnetic beads (Dynabeads M270 Streptavidin; Thermo Fisher Scientific) were recorded for 20 s and analyzed using 3DPT using 15 polynomials during phase calibration and 15 reference images during tracking. Drift and mechanical vibrations were removed following the approach described in Materials and Methods, and a typical trace was plotted in the *x* (*a*), *y* (*b*), and *z* (*c*) directions. Some residual drift that was present in all dimensions indicated imperfect immobilization of the beads. The distribution of the coordinates yielded an SD of $\sigma_{xy} = 0.6$ nm in the *x* and the *y* directions and $\sigma_z = 1.6$ nm in the *z* direction. (*d*) 25 native chromatin molecules, assembled in vivo (53), were stretched and unfolded with MT. Although the complexes were heterogeneous, they unfolded in three distinct transitions [63]. The last transition, in which the last singe turn of DNA unwraps from the histone core, takes place above ±5 pN (for native chromatin) and is recognized by its stepwise nature. The inset show a two times magnification and a 25 nm scale bar, highlighting the accuracy with which these steps can be resolved. (*e*) The step size distribution was plotted in a histogram (bin width = 12 bp) and fitted with a triple Gaussian. The histogram contained data from 111 curves from which a selection was plotted in Fig. 5 *b*. The step size was easily resolved with our approach and measured 77 ± 1 bp, taking into account double and triple simultaneous steps.

with lower spatial frequencies such as light gradients did decrease the accuracy of the calculated *z* coordinate (Fig. 5 *c*). Astigmatism up to 15% resulted in moderate increases of the error (Fig. 4 *d*). Larger astigmatism was more problematic. However, such distortions should not occur in properly designed microscopes.

We also quantified the robustness of the tracking algorithm when the diffraction pattern was not fully contained within the ROI. Because there is always a tradeoff between the size of the analyzed ROI and computation speed, it is in the interest of increasing throughput to reduce the ROI as much as possible. Moreover, restricting the ROI reduces the probability that other beads enter the ROI, which hampers accurate tracking. We observed that tracking accuracy was not affected until the bead was shifted by more than 30% out of the ROI (Fig. 4 *e*). In conclusion, our simulations showed that 3DPT was robust against many types of typical aberrations.

Finally, the performance of 3DPT was demonstrated experimentally by simultaneous measurement of the three-dimensional position of 10 immobilized 2.8 μm diameter paramagnetic beads. Drift and mechanical vibrations, which dominated our measurements, as reflected in the PSDs (Fig. 2 *j*), were characterized, isolated, and removed, following the approach described in Materials and Methods. A representative time trace in three dimensions, which is shown in Fig. 6, *a–c*, yielded an SD in the *x* and *y* directions of 0.3 nm and in the *z* direction of 1.6 nm, roughly matching the simulated values.

As an illustration of an application, we implemented our tracking algorithm to measure the unfolding of native chromatin, shown in Fig. 6 *d* (experimental details in (53)). Native chromatin is an example of a highly heterogeneous sample, in which many molecules need to be measured to extract common features. We used 3DPT to measure the step size of the unwrapping of DNA from the histone core, using the method of the step size described by Kaczmarczyk (56) and summarized in Materials and Methods. As expected, 3DPT accurately revealed the characteristic 77 bp step size, corresponding to ~25 nm, shown in Fig. 6 *e*. Although the molecules were heterogeneous in composition and unfolded accordingly, the width of the individual steps occurring at forces above 5 pN could easily be resolved.

DISCUSSION

Single-molecule biophysical techniques frequently employ bead tracking for the mechanical characterization of biomolecules. Here, we introduced 3DPT, a robust and non-iterative bead-tracking algorithm for holographic imaging. It makes use of the circular symmetry of a holographic image of a bead and, similar to lock-in techniques, selects a single spatial frequency for image analysis. Next to directly producing the *xy* position, the height information

is captured in a single parameter: the phase of the wave front of the diffraction pattern, which can be converted to a z coordinate using calibration before the experiment. 3DPT was lightweight, robust against common aberrations, and yielded nanometer accuracy in three dimensions. We have implemented this algorithm in NI LabVIEW 2014 on a 10 core 2.8 GHz CPU and could track up to 10,000 diffraction patterns per second captured within a ROI of 100 $\times$ 100 pixels. 3DPT is robust against many common image artifacts and can yield nanometer accuracy despite suboptimal imaging conditions.

One could alternatively use 2D cross correlation with a set of experimentally obtained reference images, which would include unknown variables such as optical aberrations and bead-dependent properties. Because holographic images contain a range of spatial frequencies, the phase of such a cross correlation is ill-defined, and therefore, only the amplitude can be used to determine the height, analogous with the 1D LUT method. Such an algorithm, however, as compared to 3DPT, requires more 2D FFTs per calculation, making it much slower, and would also propagate errors in the reference images. We found that 3DPT is not only faster but also more robust.

Because of drift and mechanical vibrations in our setup, we did not test whether 3DPT could resolve nanometer steps, as was demonstrated before (51). Nevertheless, 25 nm steps, resulting from unwrapping single nucleosome, were easily resolved. From PSD analysis, as well as unfiltered time traces, it is clear that 1 nm is close to the limit of the accuracy of our experiments at 30 Hz bandwidth. 1 Hz averaging should be able to resolve nanometer changes in bead position. In the lateral direction, 3DPT performs an order of magnitude better. For many applications other than single-molecule force spectroscopy, the lateral resolution is as important as accuracy in the z direction.

Traditional LUT-based bead-tracking algorithms proved to be faster than the more robust 3DPT, and, when imaging conditions can be optimized, they appear to yield more accurate tracking results. Computing the radial intensity profile is the most time-consuming step in LUT bead-tracking algorithms, and transferring this step to GPU can increase the processing speed (50). We nevertheless do not expect a significant gain in speed by implementing 3DPT in GPU because the current computation times are smaller than the time it takes for transferring the images from CPU to GPU. Avoiding computation of the radial intensity profile makes the tracking more robust: small errors in xy position, as well as imaging aberrations, introduce large changes in this profile as compared to a reference image. In 3DPT, these artifacts predominantly reduce the amplitude of the cross correlation but have little effect on the position or the phase.

Because the computer-generated reference images that are used in 3DPT have a known center and lack noise and other artifacts, the algorithm is very robust. We therefore expect that 3DPT may be used for other applications than the tracking of spherical colloidal particles. This, however, should be tested for each particular application. In addition to bead tracking, we also used a cross correlation with reference images for autofocusing and for initial recognition of beads, relieving the operator from manually selecting beads. For multiplexed high-throughput applications, this can be a key advantage because the microscopy can be fully automated.

We could not obtain good fits of our super-paramagnetic beads to LMST, resulting in large tracking errors, typically hundreds of nanometers, which we tentatively attributed to the heterogeneous composition of the beads. This suggests that LMST fitting is a more viable option for, for example, TPM, AFS, and OT, which do not require magnetic beads. Though fitting imprecisions did not significantly affect the resulting position accuracy in the xy direction, it was detrimental for tracking in the z direction. Fits only converged in a limited range, especially in the case of the 2.8 μm beads. For MT, LMST fitting therefore may not only impede real-time processing, but it may also be inadequate for applications that require more than a several-micrometer range.

In previous work, the Grier group used a laser to create holographic images (41). Most biophysical single-molecule studies (including ours), however, used a collimated LED to illuminate the sample. Because of the limited spatial coherence of an LED, the images do not feature speckle patterns, which were subtracted in studies using LMST fitting (61). The limited coherence also reduces the range of the diffraction pattern, and laser illumination in combination with image background subtraction may further improve the accuracy of 3DPT by generating more contrast in the holographic image.

Multiplexing becomes increasingly important in single-molecule biophysics as more complex and more heterogeneous samples are investigated. A good example is our previous study, in which we performed force spectroscopy on natively assembled chromatin fibers (53). Multiplexing allowed us to pick out hundreds of chromatin fibers that each unfolded differently, reflecting variations in composition and folding. Another example is a study on RNA polymerase pausing, in which multiplexing served to observe sufficient rare events to analyze the statistics (62). Because 3DPT can easily replace traditional radial profile comparisons with LUTs, it may be adopted by many experimentalists.

Overall, its easy implementation, robustness, and nanometer accuracy over a wide z range makes 3DPT an ideal method for multiplexed particle tracking applications. It may improve all single-molecule techniques that rely on bead tracking such as MT, TPM, AFS, and OT. By enhancing their throughput, it will help to realize one of the truly unique promises of single-molecule biophysics: detecting rare events in a large population of molecules with unprecedented resolution.

SUPPORTING MATERIAL

Supporting Material can be found online at https://doi.org/10.1016/j.bpj.2020.01.015.

AUTHOR CONTRIBUTIONS

T.B.B. and J.v.N. conceived the study. T.B.B., N.H., and J.v.N. designed and performed the research. T.B.B. and J.v.N. analyzed data, performed the simulations, and developed the mathematical framework. N.H. measured and analyzed the chromatin data.

ACKNOWLEDGMENTS

The authors thank Artur Kaczmarczyk for valuable discussions during the development of the algorithm.

This work is part of the research program VICI with project number 680-47-616, which is (partly) financed by the Dutch Research Council (NWO).

REFERENCES

1. Neuman, K. C., and A. Nagy. 2008. Single-molecule force spectroscopy: optical tweezers, magnetic tweezers and atomic force microscopy. *Nat. Methods.* 5:491–505.
2. Bustamante, C., Z. Bryant, and S. B. Smith. 2003. Ten years of tension: single-molecule DNA mechanics. *Nature.* 421:423–427.
3. Wuite, G. J., R. J. Davenport, …, C. Bustamante. 2000. An integrated laser trap/flow control video microscope for the study of single biomolecules. *Biophys. J.* 79:1155–1167.
4. Baumann, C. G., S. B. Smith, …, C. Bustamante. 1997. Ionic effects on the elasticity of single DNA molecules. *Proc. Natl. Acad. Sci. USA.* 94:6185–6190.
5. Smith, S. B., Y. Cui, and C. Bustamante. 1996. Overstretching B-DNA: the elastic response of individual double-stranded and single-stranded DNA molecules. *Science.* 271:795–799.
6. Kaczmarczyk, A., A. Allahverdi, …, J. van Noort. 2017. Single-molecule force spectroscopy on histone H4 tail-cross-linked chromatin reveals fiber folding. *J. Biol. Chem.* 292:17506–17513.
7. Meng, H., K. Andresen, and J. van Noort. 2015. Quantitative analysis of single-molecule force spectroscopy on folded chromatin fibers. *Nucleic Acids Res.* 43:3578–3590.
8. Kruithof, M., F. Chien, …, J. van Noort. 2008. Subpiconewton dynamic force spectroscopy using magnetic tweezers. *Biophys. J.* 94:2343–2348.
9. Chien, F.-T., and J. van Noort. 2009. 10 years of tension on chromatin: results from single molecule force spectroscopy. *Curr. Pharm. Biotechnol.* 10:474–485.
10. Eslami-Mossallam, B., H. Schiessel, and J. van Noort. 2016. Nucleosome dynamics: sequence matters. *Adv. Colloid Interface Sci.* 232:101–113.
11. Kaczmarczyk, A., T. B. Brouwer, …, J. van Noort. 2018. Probing chromatin structure with magnetic tweezers. *In* Nanoscale Imaging: Methods and Protocols. Y. Lyubchenko, ed. Humana Press, pp. 297–323..
12. van der Valk, R. A., J. Vreede, …, R. T. Dame. 2017. Mechanism of environmentally driven conformational changes that modulate H-NS DNA-bridging activity. *eLife.* 6:e27369.
13. Henneman, B., J. Heinsman, …, R. T. Dame. 2018. Quantitation of DNA-binding affinity using tethered particle motion. *Methods Mol. Biol.* 1837:257–275.
14. van Noort, J., S. Verbrugge, …, R. T. Dame. 2004. Dual architectural roles of HU: formation of flexible hinges and rigid filaments. *Proc. Natl. Acad. Sci. USA.* 101:6969–6974.
15. Luijsterburg, M. S., M. F. White, …, R. T. Dame. 2008. The major architects of chromatin: architectural proteins in bacteria, archaea and eukaryotes. *Crit. Rev. Biochem. Mol. Biol.* 43:393–418.
16. Brouwer, T. B., A. Kaczmarczyk, …, J. van Noort. 2018. Unraveling DNA organization with single-molecule force spectroscopy using magnetic tweezers. *Methods Mol. Biol.* 1837:317–349.
17. Strick, T. R., J.-F. Allemand, …, V. Croquette. 1996. The elasticity of a single supercoiled DNA molecule. *Science.* 271:1835–1837.
18. Strick, T., J. Allemand, …, D. Bensimon. 2000. Twisting and stretching single DNA molecules. *Prog. Biophys. Mol. Biol.* 74:115–140.
19. Meng, H., J. Bosman, …, J. van Noort. 2014. Coexistence of twisted, plectonemic, and melted DNA in small topological domains. *Biophys. J.* 106:1174–1181.
20. Kriegel, F., N. Ermann, and J. Lipfert. 2017. Probing the mechanical properties, conformational changes, and interactions of nucleic acids with magnetic tweezers. *J. Struct. Biol.* 197:26–36.
21. Brouwer, I., H. Zhang, …, M. Modesti. 2017. Human RAD52 captures and holds DNA strands, increases DNA flexibility, and prevents melting of duplex DNA: implications for DNA recombination. *Cell Rep.* 18:2845–2853.
22. Brouwer, I., T. Moschetti, …, E. J. Peterman. 2018. Two distinct conformational states define the interaction of human RAD51-ATP with single-stranded DNA. *EMBO J.* 37:e98162.
23. Brouwer, I., G. Sitters, …, G. J. Wuite. 2016. Sliding sleeves of XRCC4-XLF bridge DNA and connect fragments of broken DNA. *Nature.* 535:566–569.
24. Sitters, G., D. Kamsma, …, G. J. Wuite. 2015. Acoustic force spectroscopy. *Nat. Methods.* 12:47–50.
25. Kamsma, D., R. Creyghton, …, E. J. Peterman. 2016. Tuning the music: acoustic force spectroscopy (AFS) 2.0. *Methods.* 105:26–33.
26. Kamsma, D., and G. J. L. Wuite. 2018. Single-molecule measurements using acoustic force spectroscopy (AFS). *Methods Mol. Biol.* 1665:341–351.
27. Song, D., B. Mousley, …, A. C. Price. 2015. Tethered particle motion with single DNA molecules. *Am. J. Phys.* 83:418–426.
28. van der Valk, R. A., J. Vreede, …, R. T. Dame. 2014. Genomic looping: a key principle of chromatin organization. *J. Mol. Microbiol. Biotechnol.* 24:344–359.
29. Sun, Y.-L., Z.-P. Luo, …, K.-N. An. 2004. Stretching type II collagen with optical tweezers. *J. Biomech.* 37:1665–1669.
30. Shayegan, M., N. Rezaei, …, N. R. Forde. 2013. Probing multiscale mechanics of collagen with optical tweezers. *In* Optical Trapping and Optical Micromanipulation X. K. Dholakia and G. C. Spalding, eds. International Society for Optics and Photonics, p. 88101P.
31. Rezaei, N., B. P. B. Downing, …, N. R. Forde. 2011. Using optical tweezers to study mechanical properties of collagen. *In* Photonics North 2011. R. Kashyap, M. Têtu, and R. N. Kleiman, eds. International Society for Optics and Photonics, p. 80070K.
32. Kamsma, D., P. Bochet, …, T. Rose. 2018. Single-cell acoustic force spectroscopy: resolving kinetics and strength of T cell adhesion to fibronectin. *Cell Rep.* 24:3008–3016.
33. van der Horst, A., and N. R. Forde. 2010. Power spectral analysis for optical trap stiffness calibration from high-speed camera position detection with limited bandwidth. *Opt. Express.* 18:7670–7677.
34. van der Horst, A., and N. R. Forde. 2008. Calibration of dynamic holographic optical tweezers for force measurements on biomaterials. *Opt. Express.* 16:20987–21003.
35. Yu, Z., D. Dulin, …, N. H. Dekker. 2014. A force calibration standard for magnetic tweezers. *Rev. Sci. Instrum.* 85:123114.
36. Daldrop, P., H. Brutzer, …, R. Seidel. 2015. Extending the range for force calibration in magnetic tweezers. *Biophys. J.* 108:2550–2561.

37. Lansdorp, B. M., S. J. Tabrizi, …, O. A. Saleh. 2013. A high-speed magnetic tweezer beyond 10,000 frames per second. *Rev. Sci. Instrum.* 84:044301.

38. Ribeck, N., and O. A. Saleh. 2008. Multiplexed single-molecule measurements with magnetic tweezers. *Rev. Sci. Instrum.* 79:094301.

39. De Vlaminck, I., T. Henighan, …, C. Dekker. 2011. Highly parallel magnetic tweezers by targeted DNA tethering. *Nano Lett.* 11:5489–5493.

40. De Vlaminck, I., T. Henighan, …, C. Dekker. 2012. Magnetic forces and DNA mechanics in multiplexed magnetic tweezers. *PLoS One.* 7:e41432.

41. Lee, S.-H., Y. Roichman, …, D. G. Grier. 2007. Characterizing and tracking single colloidal particles with video holographic microscopy. *Opt. Express.* 15:18275–18282.

42. Ghosh, R. N., and W. W. Webb. 1994. Automated detection and tracking of individual and clustered cell surface low density lipoprotein receptor molecules. *Biophys. J.* 66:1301–1318.

43. Lee, G. M., A. Ishihara, and K. A. Jacobson. 1991. Direct observation of brownian motion of lipids in a membrane. *Proc. Natl. Acad. Sci. USA.* 88:6274–6278.

44. Brown, L. G. 1992. A survey of image registration techniques. *ACM Comput. Surv.* 24:325–376.

45. Kusumi, A., Y. Sako, and M. Yamamoto. 1993. Confined lateral diffusion of membrane receptors as studied by single particle tracking (nanovid microscopy). Effects of calcium-induced differentiation in cultured epithelial cells. *Biophys. J.* 65:2021–2040.

46. Gelles, J., B. J. Schnapp, and M. P. Sheetz. 1988. Tracking kinesin-driven movements with nanometre-scale precision. *Nature.* 331:450–453.

47. Cheezum, M. K., W. F. Walker, and W. H. Guilford. 2001. Quantitative comparison of algorithms for tracking single fluorescent particles. *Biophys. J.* 81:2378–2388.

48. Gosse, C., and V. Croquette. 2002. Magnetic tweezers: micromanipulation and force measurement at the molecular level. *Biophys. J.* 82:3314–3329.

49. van Loenhout, M. T. J., J. W. J. Kerssemakers, …, C. Dekker. 2012. Non-bias-limited tracking of spherical particles, enabling nanometer resolution at low magnification. *Biophys. J.* 102:2362–2371.

50. Cnossen, J. P., D. Dulin, and N. H. Dekker. 2014. An optimized software framework for real-time, high-throughput tracking of spherical beads. *Rev. Sci. Instrum.* 85:103712.

51. Dulin, D., T. J. Cui, …, N. H. Dekker. 2015. High spatiotemporal-resolution magnetic tweezers: calibration and applications for DNA dynamics. *Biophys. J.* 109:2113–2125.

52. Chien, F. T., and T. van der Heijden. 2014. Characterization of nucleosome unwrapping within chromatin fibers using magnetic tweezers. *Biophys. J.* 107:373–383.

53. Hermans, N., J. J. Huisman, …, J. van Noort. 2017. Toehold-enhanced LNA probes for selective pull down and single-molecule analysis of native chromatin. *Sci. Rep.* 7:16721.

54. Mie, G. 1908. Beiträge zur Optik trüber Medien, speziell kolloidaler Metallösungen. *Ann. Phys.* 330:377–445.

55. Shen, J. 2005. Algorithm of numerical calculation on Lorentz Mie theory. *PIERS Online.* 1:691–694.

56. Kaczmarczyk, A. 2019. Nucleosome Stacking in Chromatin Fibers: Probed with Single-Molecule Force- and Torque-Spectroscopy. Leiden University, Leiden, the Netherlands.

57. Pritt, M. D., and J. S. Shipman. 1994. Least-squares two-dimensional phase unwrapping using FFT's. *IEEE Trans. Geosci. Remote Sens.* 32:706–708.

58. Pritt, M., and D. Ghiglia. 1998. Two-Dimensional Phase Unwrapping: Theory, Algorithms, and Software. Wiley, Hoboken, NJ.

59. Ghiglia, D. C., and L. A. Romero. 1994. Robust two-dimensional weighted and unweighted phase unwrapping that uses fast transforms and iterative methods. *J. Opt. Soc. Am. A Opt. Image Sci. Vis.* 11:107.

60. Bone, D. J. 1991. Fourier fringe analysis: the two-dimensional phase unwrapping problem. *Appl. Opt.* 30:3627–3632.

61. Cheong, F. C., B. J. Krishnatreya, and D. G. Grier. 2010. Strategies for three-dimensional particle tracking with holographic video microscopy. *Opt. Express.* 18:13563–13573.

62. Dulin, D., I. D. Vilfan, …, N. H. Dekker. 2015. Elongation-competent pauses govern the fidelity of a viral RNA-dependent RNA polymerase. *Cell Rep.* 10:983–992.

Biophysical Journal, Volume *118*

Supplemental Information

Multiplexed Nanometric 3D Tracking of Microbeads Using an FFT-Phasor Algorithm

Thomas B. Brouwer, Nicolaas Hermans, and John van Noort

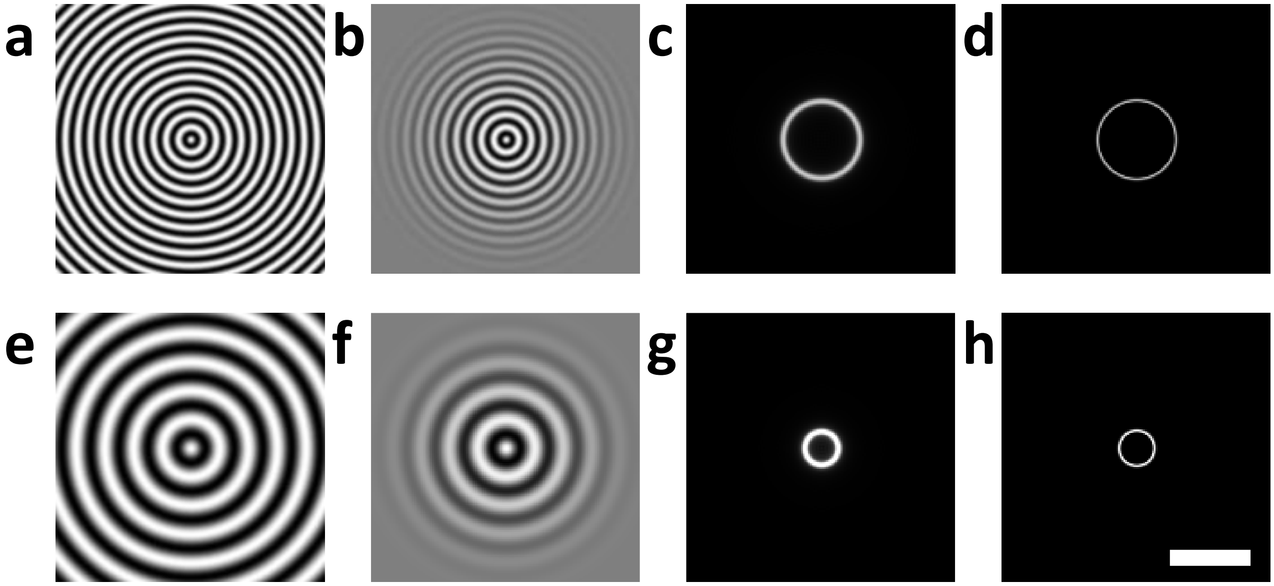

Figure S1) Reference images and corresponding band-pass filters for 3DPT. A reference image containing a single spatial frequency of period $k_A = 7$ pix (a) was filtered by a Hamming filter (b). The reference image was transformed into Fourier space (c), and filtered by a frequency band-pass filter (d). Panel e-h depicted a reference image containing a spatial frequency of period $k_B = 16$ pix. Scale bar: 3 µm.

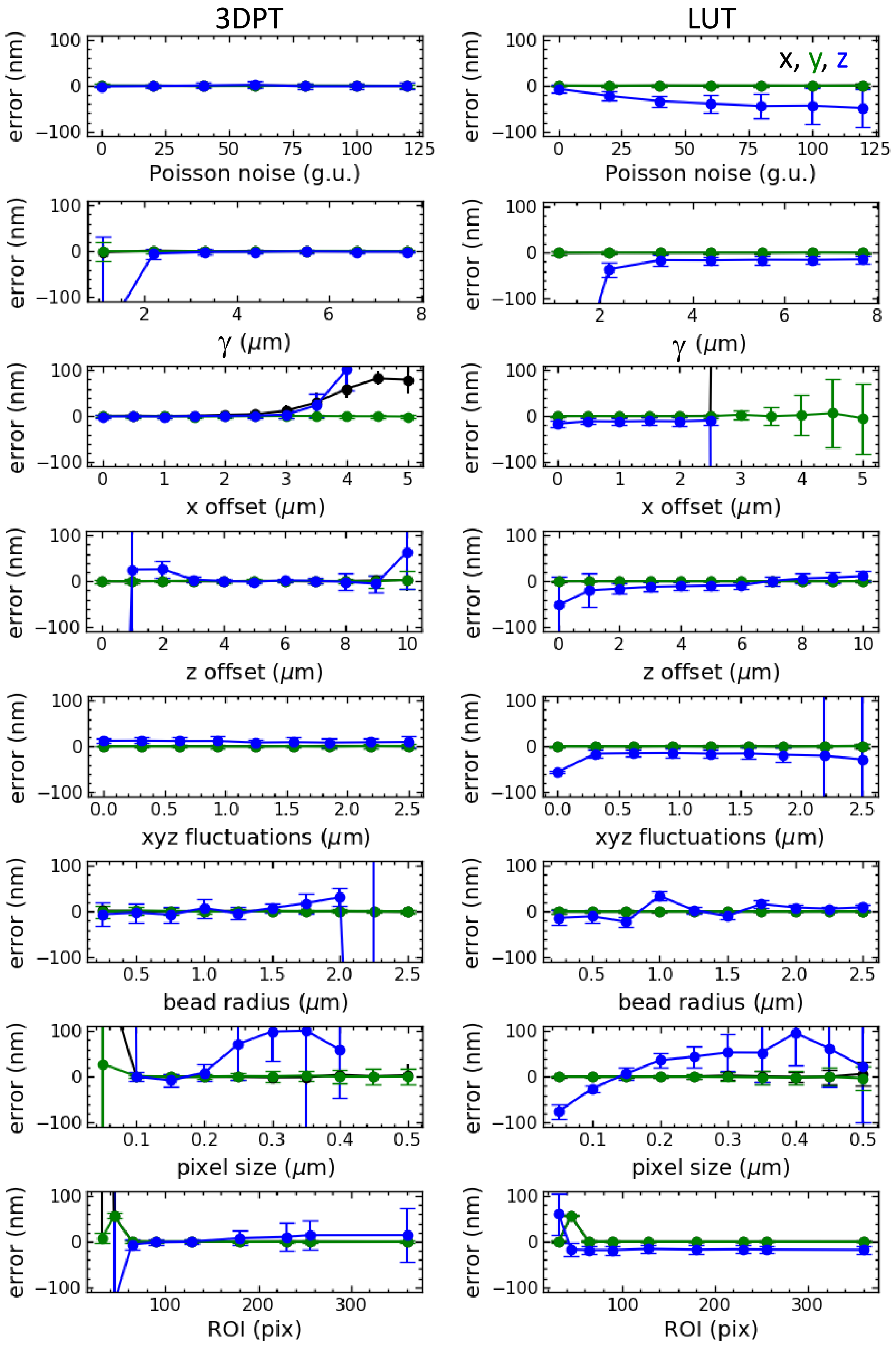
3DPT
LUT
x, y, z
error (nm)
100
0
−100
Poisson noise (g.u.)
γ (μm)
x offset (μm)
z offset (μm)
xyz fluctuations (μm)
bead radius (μm)
pixel size (μm)
ROI (pix)

Figure S2) Comparison between 3DPT and LUT accuracy. The accuracies as a function of several parameters that define a tracking experiment were computed using LMST simulations. We used the following standard parameters: ROI size = 100 pixels, pixel size 0.11 µm, number of radial profiles = 64, z-range for calibration was 0 to 15 µm, X and Y offset = 0 µm, Poisson noise = 0 gray scale units. A standard tracking simulation consisted of 256 frames, bead diameter = 1 µm, a linear z-ramp from 4 to 7 µm, X and Y offset = 0 µm, Poisson noise = 5 gray scale units and X, Y, and Z positions that were drawn from a normal distribution with a width of 100 nm. Points represent median difference between input coordinates and computed coordinates. Error bars represent inter quartile ranges of the fluctuations within a tracking simulation. Means and standard deviations were disproportionally affected by rare outliers in the LUT tracking. X and Y data generally overlap and demonstrate the reproducibility of these simulations. Graphs on the left represent 3DPT results, graphs on the right LUT-tracking results.

Poisson noise hardly affected 3DPT results, but introduced a systematically smaller bead height using LUT tracking. Coherence length of the illumination source and NA of the objective were effectively included in the width of the Hamming filter γ, and did not affect tracking accuracy of 3DPT when larger than 2 µm. For LUT, a similar trend was found, but the z coordinate was again underestimated. Offsets in the imaging plane did not affect accuracy up to 3 µm for 3DPT, and was inhibiting LUT tracking. In the z direction, 3DPT was accurate between 3 and 9 µm above focus. Here LUT tracking appeared robust over a larger range, but accuracy was a bit decreased. Simultaneous fluctuations in all 3 directions, mimicking more realistic experiments, did not affect 3DPT accuracy within 2.5 µm, whereas LUT tracking produced large variations in accuracy beyond 2 µm. For bead radii larger than 2 µm we observed reduced accuracy in 3DPT, which resulted from a more intricate diffraction pattern in close vicinity of the bead (data not shown). To remedy this, we computed images at 8 µm above focus, rather than 4 µm, which resulted in less convolved holograms. As a result 3DPT performed well again, up to beads with a radius larger than 2 µm. LUT tracking proved to be more flexible in terms of bead size, though slightly less accurate. Image magnification also affected tracking accuracy for 3DPT. This is not surprising as the spatial frequencies in the holographic image directly scale with magnification. The chosen frequencies were optimized for a magnification corresponding to 0.11 µm per pixel. Larger magnifications yielded poorer accuracy. However, this can be remedied by scaling the spatial frequencies in the reference images accordingly. Somewhat surpassingly, the LUT method also performed less with increasing magnification. This can probably be attributed to the reduced number of fringes that are captured in a fixed sized ROI. Finally, ROI sizes above 64 pixels were sufficient for both methods to yield accurate results. Note however that 3DPT became less accurate for very large ROIs, presumably because of the increased influence of Poisson noise when more pixels are included. Apparently LUT tracking is better at averaging out these fluctuations.

Overall, both methods can achieve similar accuracies over a large range of parameters. 3DPT appeared more robust, yielding < 10 nm accuracy over a broader range of parameters, and featured smaller systematic errors.

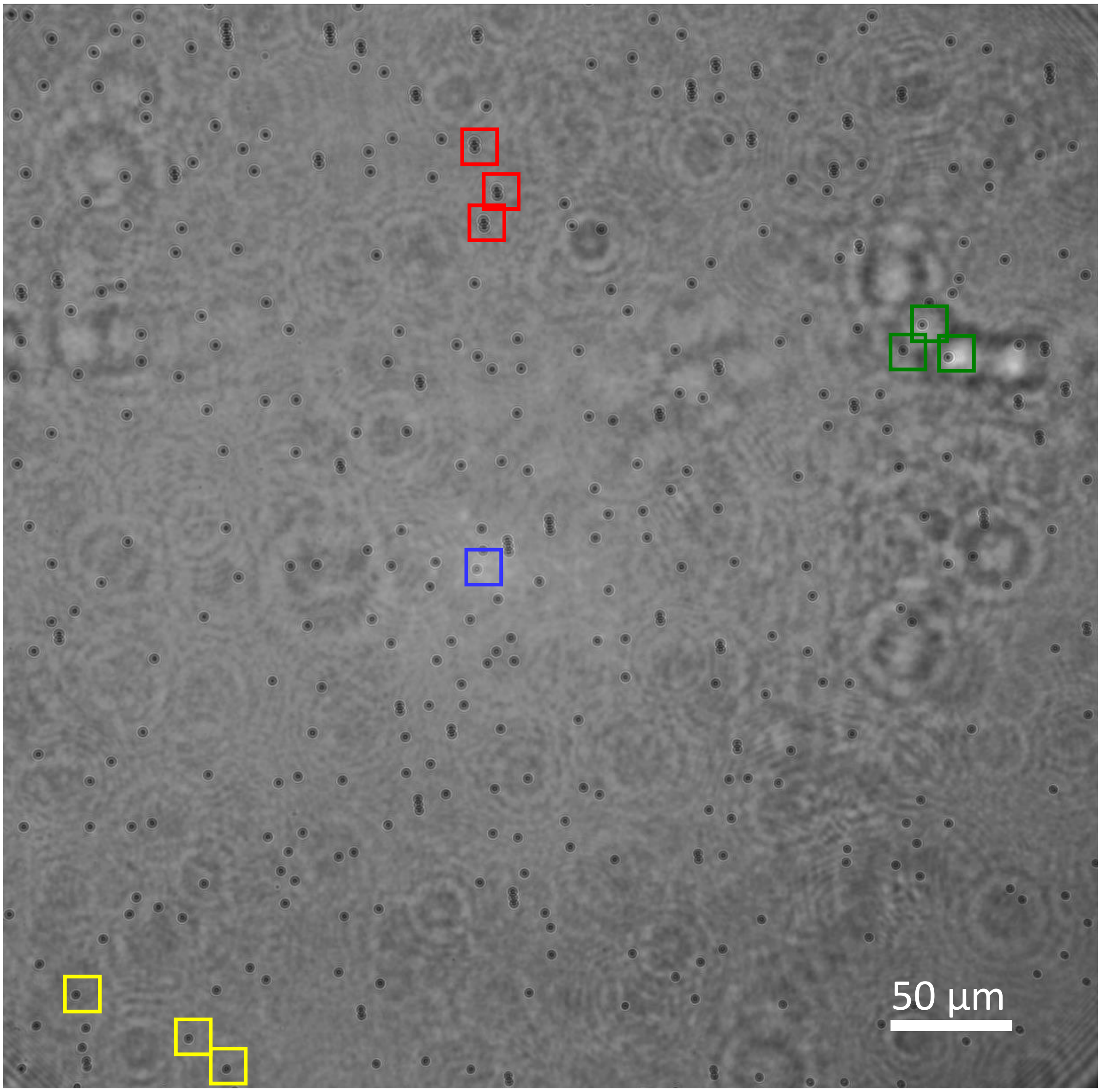

Figure S3) Typical image artefacts in large area imaging. This image shows a full 4096x4096 pixels field of view of tethered beads in our MT microscope. Though holographic images of the beads are well resolved throughout the image, inhomogeneous illumination and image distortions deform the bead images. In particular spherical aberrations dominate at the edges, leading to elongated diffraction patterns (yellow). The center of the field of view was somewhat over exposed, reducing the contrast in the central ROIs (blue). Loose beads, well above the focus plane, obstruct illumination and cause a gradient in the background (green). 3DPT is quite tolerant for such distortions (see figure 5 in the main text). Aggregated beads (red) however, not only affect tracking, but also result in increased forces that are applied to the tether. These beads should be discarded from further analysis. Surprisingly, bead aggregation were not easily identified from 3DPT traces, which were remarkably accurate and error free for small aggregates and should therefore be identified directly in the image. Overall, 3DPT could accurately track all beads in this image.
